# Increased Mast Cell Density and Airway Responses to Allergic and Non-Allergic Stimuli in a Sheep Model of Chronic Asthma

**DOI:** 10.1371/journal.pone.0037161

**Published:** 2012-05-14

**Authors:** Joanne Van der Velden, Donna Barker, Garry Barcham, Emmanuel Koumoundouros, Kenneth Snibson

**Affiliations:** 1 Centre for Animal Biotechnology, Veterinary Science, University of Melbourne, Parkville, Australia; 2 Department of Pharmacology, University of Melbourne, Parkville, Australia; 3 School of Engineering, University of Melbourne, Parkville, Australia; Leiden University Medical Center, Netherlands

## Abstract

**Background:**

Increased mast cell (MC) density and changes in their distribution in airway tissues is thought to contribute significantly to the pathophysiology of asthma. However, the time sequence for these changes and how they impact small airway function in asthma is not fully understood. The aim of the current study was to characterise temporal changes in airway MC density and correlate these changes with functional airway responses in sheep chronically challenged with house dust mite (HDM) allergen.

**Methodology/Principal Findings:**

MC density was examined on lung tissue from four spatially separate lung segments of allergic sheep which received weekly challenges with HDM allergen for 0, 8, 16 or 24 weeks. Lung tissue was collected from each segment 7 days following the final challenge. The density of tryptase-positive and chymase-positive MCs (MC_T_ and MC_TC_ respectively) was assessed by morphometric analysis of airway sections immunohistochemically stained with antibodies against MC tryptase and chymase.

MC_T_ and MC_TC_ density was increased in small bronchi following 24 weeks of HDM challenges compared with controls (P<0.05). The MC_TC_/MC_T_ ratio was significantly increased in HDM challenged sheep compared to controls (P<0.05). MC_T_ and MC_TC_ density was inversely correlated with allergen-induced increases in peripheral airway resistance after 24 weeks of allergen exposure (P<0.05). MC_T_ density was also negatively correlated with airway responsiveness after 24 challenges (P<0.01).

**Conclusions:**

MC_T_ and MC_TC_ density in the small airways correlates with better lung function in this sheep model of chronic asthma. Whether this finding indicates that under some conditions mast cells have protective activities in asthma, or that other explanations are to be considered requires further investigation.

## Introduction

Asthma is a chronic inflammatory disease of the airways, which is characterised by airway inflammation, reversible airflow limitation and hyperresponsive airways. Mast cells (MCs) are important mediator-secretor cells in allergic reactions and are thought to play a major role in the pathophysiology of asthma. Human MCs can be divided into two major groups based on their protease content. There are those that express tryptase but not chymase (MC_T_) and those that express tryptase and chymase (MC_TC_). Although these two MC phenotypes are known to differ in their tissue distribution and functional characteristics in healthy lungs, their relative contribution to the asthmatic phenotype is not fully understood.

It is widely established that MCs play a key role in the pathogenesis of asthma. In particular, tryptase released by MCs has been shown to induce airway inflammation and airway hyperresponsiveness (AHR) [1], [2], [3], while MC chymase promotes the recruitment of eosinophils and neutrophils to airway tissues [4]. Furthermore, increased MC numbers in the airway smooth muscle (ASM) of asthmatic patients correlates strongly with AHR [5].

Investigations of airway changes in asthmatic patients predominantly rely on airway tissue from bronchial biopsies collected from the large proximal airways, which offers little or no information on changes in the small peripheral airways. For mast cell-linked research, large animal models such as primates and sheep have been identified as being good models for chronic asthma [6], [7], [8], [9] because, in contrast to rodent models, they have MC_T_ and MC_TC_ phenotypes distributed throughout the tracheobronchial tree [10]. In particular, sheep have a similar density of mast cells in the small airways to that reported for humans [11] making the ovine model relevant for studying the human disease.

In a recent study of small airway function in sheep, we characterized progressive changes in peripheral airway responses to chronic house dust mite (HDM) challenges using a segmental challenge technique [12]. We found that there was a decline in peripheral airway function with HDM exposure and that airway responses to both allergic and non-allergic stimuli were localized to specific treated areas of the lung. We now present an analysis of progressive changes in mast cell density with increasing exposure to HDM in these sheep. We also investigate the relationship between mast cell numbers and peripheral airway function in this large animal model of asthma.

## Methods

### Ethics statement

All experimental animal procedures and the collection of tissues/cells were approved by the Animal Experimentation Ethics Committee of the University of Melbourne (approval no. 06128).

### Challenge Protocol

To investigate progressive changes in MC density we studied archived lung tissue collected from a segmental challenge protocol as previously described [12]. Briefly fourteen female Merino-cross sheep (6 months) were sensitized to HDM (*Dermatophagoides pteronyssins*; CLS, Melbourne, Australia) via 3 subcutaneous injections of HDM-alum, delivered 2 weeks apart. Pre- and post-immunisation HDM-specific serum IgE was assessed by enzyme-linked immunosorbent assay (ELISA) and sheep with at least a 2-fold increase were considered atopic for HDM-challenges (n = 7) [13]. Four spatially separate lung segments were identified and challenged weekly with HDM for different durations. A fibre-optic bronchoscope was for the delivery of solubilized HDM (1 mg in 5 mL phosphate buffered saline [PBS]). The commencement of challenges in each segment was staggered so that at the end of the challenge regime the right apical, right medial, right caudal and left caudal segments received 0, 8, 16 or 24 weeks of allergen challenges respectively, with the last challenge in each segment occurring 7 days prior to euthanasia. To ensure that there were no natural differences in mast cell density between segments, a separate group of control sheep that did not receive any HDM treatment was used (n = 7).

### Bronchoalveolar lavage collection

Bronchoalveolar lavage (BAL) fluid was collected as previously described [12]. The total number of cells collected was determined with a haemocytometer. Differential leukocyte counts were performed on cytospots of BAL cells stained with Haem Kwik Differential Stains (HD Scientific, Wetherill Park, Australia) to identify leukocytes.

### Lung tissue collection, immunohistochemistry and morphometric analysis

Sheep were euthanized by intravenous barbiturate overdose. Lung segments were inflated with optimal cutting temperature compound (ProSciTech, Thuringoura, Australia) diluted in PBS (1∶1). Mast cells were identified in a serial set of airway sections were stained separately with rat anti-ovine tryptase (1∶25), rat anti-ovine chymase antibodies (1∶25) (kindly provided by Prof Hugh Miller, University of Edinburgh, UK). Eosinophils were identified in sections stained with mouse anti-ovine galectin-14 (clone EL1.2; Centre for Animal Biotechnology, Parkville, Australia). First, sections were blocked for endogenous peroxidase and then incubated with primary antibody for two hours. After washing sections were incubated with swine anti-rat HRP conjugated secondary antibody (1∶100; Dako, Kingsgrove, Australia). Sections were then washed and a peroxidase based detection system was used for visualization [11]. Specificity of staining was confirmed by omission of the primary antibody.

All morphometric measurements of the airways were made using digital image analysis (Image-Pro Plus, Media Cybernetics, version 4.1.0.0). The area of the airway wall was determined by the area enclosed by the outer perimeter of the basement membrane (BM) and the outer perimeter of the airway wall as previously described [14]. Two small cartilaginous bronchi (approximately 1–2 mm diameter) from each segment in each sheep were examined and the two values averaged. Results are expressed as the number of positive cells per mm^2^ of airway wall. Collagen and ASM content was measured on sections stained with a Masson's Trichrome stain kit (Sigma-Aldrich, Australia), as previously described [11]. Results are expressed as the area of collagen or ASM per mm BM. All sections were scored by a single observer who was blind to the experimental group and lung segment.

### Lung function correlations

Segmental lung function indices were taken from a separate study performed on these sheep [12] and used to determine the relationship between MC density and functional airways changes.

### Data analysis

Statistical analysis was performed with GraphPad Prism software (version 4.01, GraphPad, San Diego, CA, USA). Mast cell density values reported are the mean ± standard error. Mann-Whitney U tests compared mast cell density between the control and HDM-challenge groups. Correlations were assessed by the Spearman correlation coefficient (r_s_). A P value of <0.05 was taken as significant.

## Results

### Mast cell density

**Figure 1:**
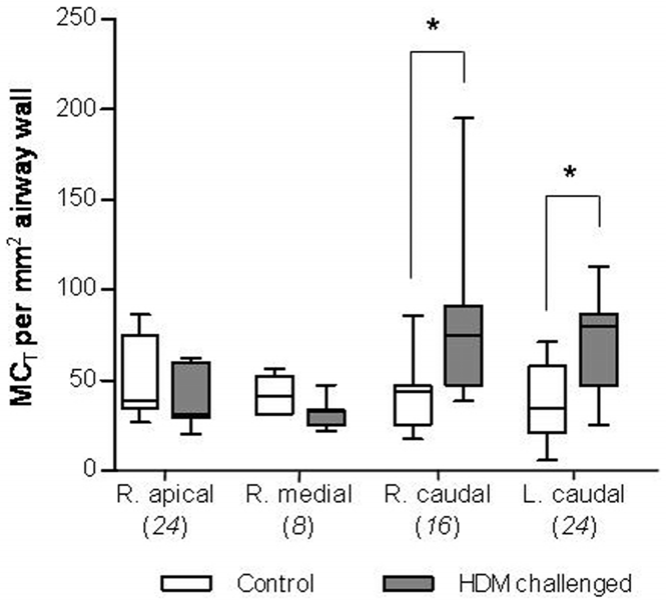
Tryptase-positive mast cell (MC_T_) density following challenge with house dust mite (HDM) allergen. Bars show the number of MC_T_ per mm^2^ in the airway wall of bronchi in four spatially separate lung segments from control and HDM challenged groups. The number in italics indicates the number of HDM challenges each lung segment received. Line shows the mean, boxes show 25^th^ and 75^th^ percentile, whiskers represent 5^th^ to 95^th^ percentile. n = 7, *P<0.05. R – right; L – left.

**Figure 2:**
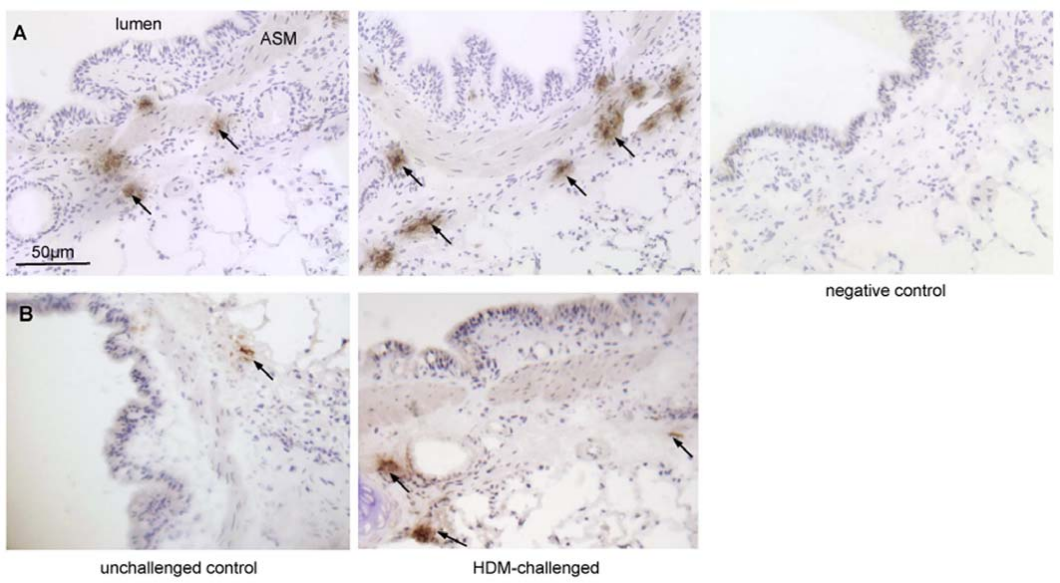
Airway sections stained with antibodies against mast cell tryptase and chymase. (a) Tryptase-positive mast cells and (b) chymase-positive mast cells. The house dust mite (HDM) treated segment received 24 weekly challenges and the control segment was untreated. Negative control shows omission of the primary antibody. Arrows indicate positive cells.

Results show that at least 16 weekly HDM challenges are required to increase the density of mast cells in the airway wall. MC_T_ density in the airway wall of bronchi was significantly increased in HDM challenged segments that received 16 and 24 challenges compared to the corresponding segments in the control group (86±21 vs. 43±8, P<0.05 and 71±11 vs. 36±8 cells/mm^2^ respectively, P<0.05; Fig. 1). The mast cells were predominantly located in the outer walls of small bronchi as illustrated in Figure 2. There was no difference in MC_T_ density in segments from HDM-challenged sheep that received 8 challenges compared to control sheep (Fig. 1). A correlation analysis revealed that MC_T_ density in bronchi was significantly positively correlated to the duration in weeks of HDM challenges (r_s_ = 0.510, P<0.01; data not shown).

**Figure 3:**
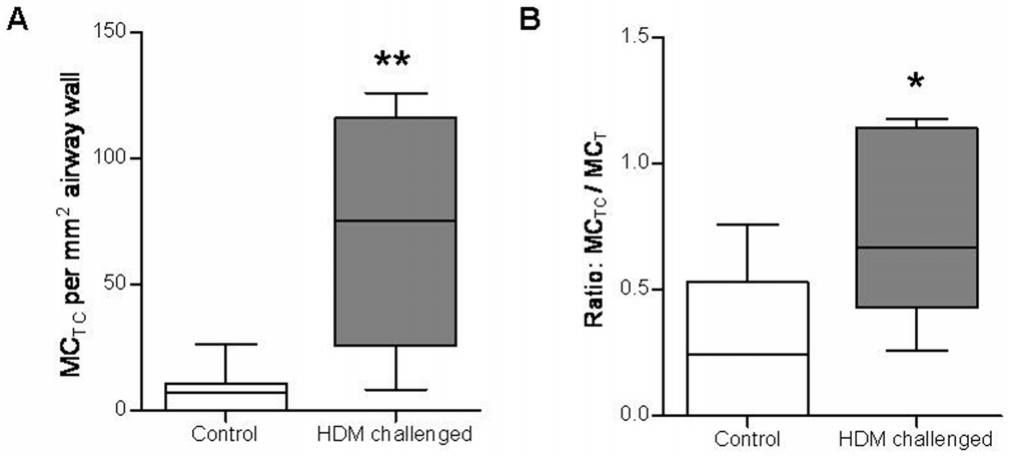
Chymase-positive mast cell (MC_TC_) density increases following chronic allergen challenge. (a) MC_TC_ per mm^2^ of airway wall (b) ratio of MC_TC_ and tryptase-positive mast cells (MC_T_). The house dust mite (HDM) treated segment received 24 weekly challenges and the control segment was untreated. Line shows the mean, boxes show 25^th^ and 75^th^ percentile, whiskers represent 5^th^ to 95th percentile. n = 7, *P<0.05, **P<0.01 compared to controls.

MC_TC_ density in the left caudal lung segment of the HDM-challenged group was significantly increased following 24 weeks of challenges compared to the control group (52±8 vs. 8±4 cells/mm^2^, P<0.01; Fig. 3a). The MC_TC_/MC_T_ ratio was significantly increased the HDM-challenged group compared to the control group (0.784±0.152 vs. 0.288±0.105, P<0.05; Fig. 3b).

### Correlation analysis between mast cells and small airway function

**Figure 4:**
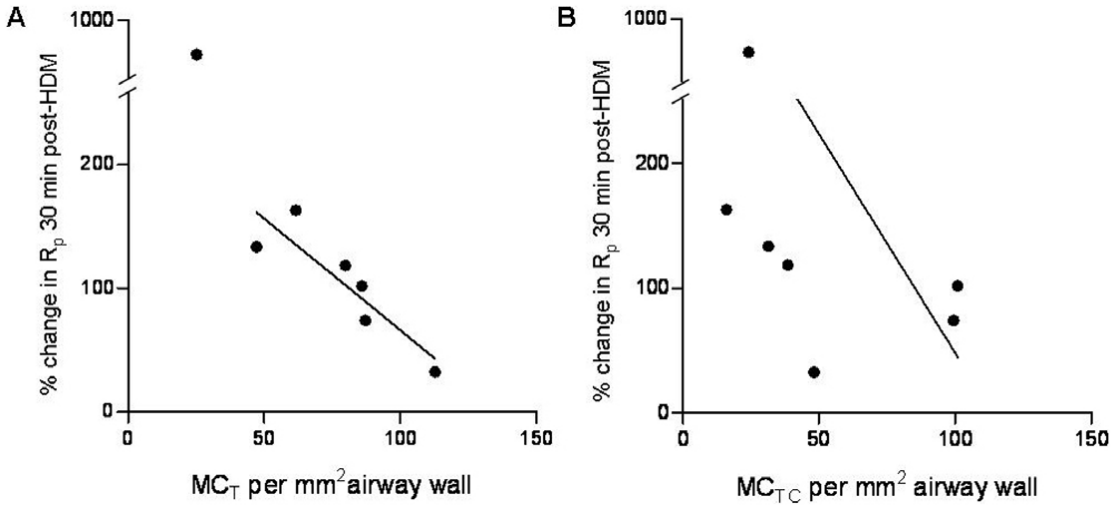
Relationship between mast cell density and peak early bronchoconstriction response. Correlation between the percentage increase in peripheral resistance (R_p_) 30 minutes post-HDM challenge and (a) MC_T_ (r_s_ = −0.89, P<0.05) and (b) MC_TC_ (r_s_ = −0.82, P<0.05).

Next we tested whether increased mast cell density in the small airways was correlated to the lung function indices reported in our previous study. MC_T_ density in bronchi after 24 weeks of challenges was significantly negatively correlated with the peak early-phase airway response (EAR); i.e. percent increase in R_p_ from baseline following a HDM challenge (r_s_ = −0.96, P<0.05; Fig. 4a). There was no correlation between MC density and EAR at any of the other time-points (data not shown). MC_TC_ density after 24 weeks of HDM challenges was also negatively correlated with early-phase responses (r_s_ = −0.82, P<0.05; Fig. 4b).

**Figure 5:**
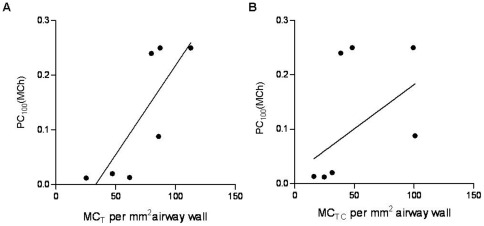
Relationship between mast cell density and airway responsiveness. Correlation between the percent dose of methacholine required to increase R_p_ by 100% (PC_100_[MCh]) from baseline and (a) MC_T_ (r_s_ = 0.92, P<0.01) and (b) MC_TC_.

The percent dose of methacholine required for a 100% increase in R_p_ (PC_100_) was positively correlated with MC_T_ (r_s_ = 0.92, P<0.01; Fig. 5a). There was also a strong trend for a positive correlation between MC_TC_ density and the dose of methacholine for PC_100_ (r_s_ = 0.74, P = 0.066; Fig. 5b). There was no correlation between MC density and airway responsiveness to methacholine at any of the other time-points. Nor was there any correlation between MC_T_ or MC_TC_ density and methacholine for PC_100_ in the untreated control sheep (r_s_ = −0.197, P = 0.662 and r_s_ = 0.060, P = 0.903 respectively).

### Airway remodeling

**Figure 6:**
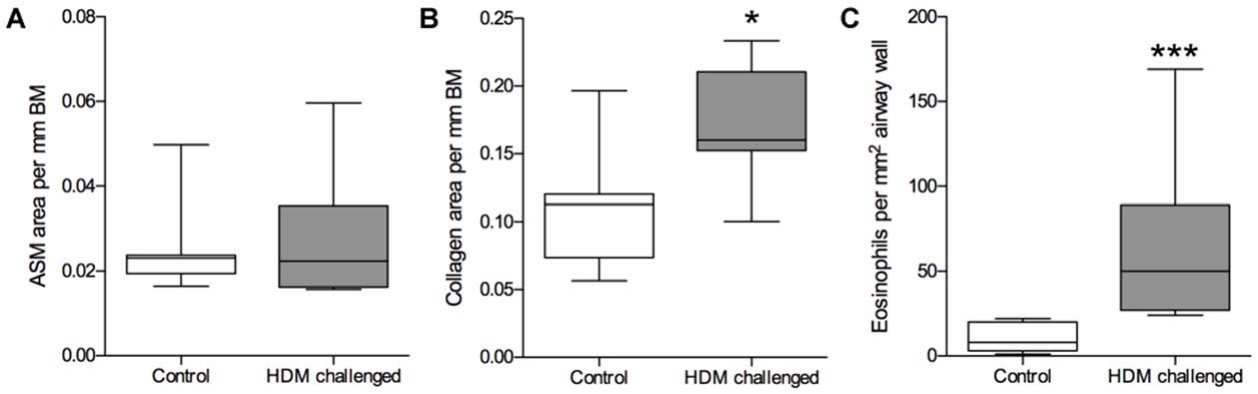
Airway remodeling and eosinophils following chronic allergen challenge. (a) Airway smooth muscle (ASM) per mm basement membrane (BM) length (b) collagen area per mm BM and (c) eosinophils per mm^2^ airway wall, in untreated control sheep and sheep challenged with house dust mite (HDM) allergen weekly for 24 weeks. Line shows the mean, boxes show 25th and 75th percentile, whiskers represent 5th to 95th percentile. n = 7, *P<0.05, ***P<0.001 compared to controls.

There was no significant difference in the amount of ASM in the lung segment that received 24 weeks of HDM compared to the corresponding segment in controls (0.029±0.005 vs. 0.025±0.004 mm^2^/BM; Fig. 6a). However, there was a significant increase in the amount of collagen (0.17±0.02 vs. 0.11±0.02 mm^2^/BM, P<0.05 Fig. 6b). There was a trend for the increase in collagen to be associated with increasing MC_TC_ density (r_s_ = 0.71, P = 0.088). The amount of airway collagen also tended to be negatively associated with EAR (r_s_ = −0.75, P = 0.066).

### Inflammatory cells

There was a significant increase in the density of eosinophils in the airway walls following 24 weeks of HDM challenges compared to the corresponding segment in controls (64.9±19.4 vs. 10.9±3.1, P<0.01; Figure 6c). There was no correlation between eosinophil density and MC_T_ or MC_TC_ concentration in HDM-challenged or unchallenged airways (r_s_ = 0.17, P = 0.713 and r_s_ = 0.57, P = 0.200; data not shown). Nor was there a correlation between eosinophil numbers and functional indices; i.e. EAR and PC_100_(MCh) in the HDM challenged segment (r_s_ = −0.071, P = 0.906 and r_s_ = 0.20, P = 0.662 respectively, data not shown). Table 1 shows that the number of eosinophils and other inflammatory cells was increased in the BAL 48 hours post-HDM at week 20 compared to the level prior to challenge. However, there was no correlation between any of the cell types examine and MC numbers, or functional indices (data not shown).

**Table 1:** Leukocyte composition of bronchoalveolar lavage cells.

	Before HDM		48 hrs post-HDM	
	Mean	SEM	Mean	SEM
Neutrophils	6.4	2.4	4.1	1.3
Eosinophils	0.4	0.3	225.8	95.4
Lymphocytes	17.1	7.9	21.4	4.9
Macrophages	152.0	36.5	141.4	34.5

Bronchoalveolar lavage (BAL) was collected from the left caudal lung segment of sheep prior to and 48 hours following HDM challenge. Differential cell counts were performed on cytospots of BAL cells. Values shown are cells (×10^5^) per mL BAL retrieved. n = 6.

## Discussion

While mast cells have long been implicated in the pathophysiology of asthma, very few studies have systematically examined the effects of mast cells on small airway function. To our knowledge this is the first time that the temporal dynamics of the mast cell population has been examined in the lungs in a large animal model of asthma. The key findings from this study are that MC_T_ and MC_TC_ density increased significantly following chronic allergen challenge and that MC density is positively correlated with the length allergen exposure and improved airway responses to allergic and non-specific stimuli.

It is noteworthy that the proportion of MCs with the chymase phenotype increases markedly with allergic challenge. Our result confirms a previous study in sheep where airways exposed to allergen have increased density of MC_TC_ [15] and is consistent the finding of increased MC_TC_ in the small airways of asthmatics [16]. It cannot be deduced from our results whether the MC_TC_ were converted from existing MC_T_ phenotype or were differentiated from newly infiltrating mast progenitor cells which originated in the bone marrow. Early studies examining mast cells developed *in vitro* from human fetal liver cells, and/or cord blood, suggest that commitment to develop as an MC_T_ or MC_TC_ phenotype may occur in MC precursor cells and that there is no evidence for conversion of MC_T_ to MC_TC_ in mature mast cells [17]. However, other *in vitro* studies provide evidence for phenotype conversion in that MC_TC_ co-cultured with human airway epithelial cells are known to convert to the MC phenotype [18], [19], suggesting that this may also be a possibility in the ovine *in vivo* experiments presented here.

Interestingly, in the present study we found a positive correlation between increased MC density and small airway function. This finding was surprising at first, given the well acknowledged central role of airway mast cells in orchestrating allergic inflammatory responses in asthma [20]. However, a close examination of the literature reveals that a positive relationship between mast cell density and lung function has been previously reported in two separate clinical studies of asthma [16], [21] and also in studies of chronic obstructive pulmonary disease (COPD) [22], [23]. In asthma in particular, MC_TC_ density in the small airways is correlated with improved lung function in mild-to-moderate and severe asthmatic subjects [16], [21]. It is unknown whether the aforementioned studies indicate that under some conditions mast cells possess protective activities in allergic airways or if other possibilities are to be considered.

One possible explanation for this correlation is that the higher concentration of MCs observed in individuals with the best airway function reflects a lower degree of degranulation and thus, a less deleterious effect. In support of this hypothesis are reports that patients who have died from asthma have a lower number of MCs in their airways compared to control cases [24] and that MC degranulation is positively associated with disease severity [25] and hence, poorer lung function.

As suggested by others, it is also conceivable that in addition to their harmful activities, MCs offer some protection against deteriorating lung function in states of disease [16]. In particular, the positive relationship between MCs and lung function could reflect an adaptive response of MCs to down-regulate some of their pro-inflammatory activities [26]. In support of this is the finding that mouse MC protease 4 (MCP-4), a chymase that is functionally homologous to human chymase, has a protective role in allergic airway inflammation [26]. Mice lacking MCP-4 were found to have significantly higher AHR and airway inflammation when sensitized and challenged with ovalbumin compared with their wild type counterparts [26].

It has been previously suggested that MCs may be able to exert protective effects by regulating ASM cells [26]. In particular, MC chymase has been shown to inhibit smooth muscle cell proliferation and thus, may prevent the associated increase in peripheral airway resistance in chronic disease [27]. It is possible that MC regulation of the ASM cells could explain the lack of an increase in ASM area in HDM challenged sheep relative to controls observed in the current study.

Another possibility is that the release of MC tryptase has some long-term protective effects for lung function by stabilising lung structure. For example, tryptase has been reported to repair and remodel damaged airway tissues in asthmatic lungs [28]. The release of tryptase into the airways has been suggested to drive fibrosis and extracellular matrix turnover [29], particularly the synthesis and secretion of type I collagen [29], potentially contributing to a stiffening of the bronchial walls and the prevention of airway collapse on expiration, thereby serving a protective function in diseased airways [30], [31]. Consistent with this hypothesis is the finding from our study that bronchial collagen deposition was significantly increased after 24 weeks of allergen challenges, and that amount airway wall collagen tended to be positively associated with MC_TC_ density.

It is important to note that therapeutic interventions against mast cell activation are likely to be important when mast cells are active in coordinating the detrimental immune response at the onset of the allergic reaction. However, the findings from the present study and that of Balzar *et al* [16], Zanini *et al* [21] and Gosman *et al* [22], that increased numbers of mast cells are associated with relatively better lung function in diseased airways, suggest that any therapeutic interventions that are active against mast cells will also need to accommodate this aspect in long-term management of airway disease.

In the present study we show a correlation between mast cell density and better airway responses to allergic and non-allergic stimuli in a large animal model. Our results support the findings of several clinical studies that report positive correlations between increased mast cell concentrations and improved lung function indices. Whether this finding indicates that under some conditions MCs have protective activities in asthma, or that other explanations are to be considered, requires further investigation.
